# Dynamics of Non-visual Responses in Humans: As Fast as Lightning?

**DOI:** 10.3389/fnins.2019.00126

**Published:** 2019-03-05

**Authors:** Abhishek S. Prayag, Sophie Jost, Pascale Avouac, Dominique Dumortier, Claude Gronfier

**Affiliations:** ^1^Lyon Neuroscience Research Center, Integrative Physiology of the Brain Arousal Systems, Waking Team, Inserm UMRS 1028, CNRS UMR 5292, Université Claude Bernard Lyon 1, Université de Lyon, Lyon, France; ^2^ENTPE, LGCB, Université de Lyon, Lyon, France

**Keywords:** light, non-visual, circadian, duration response curve, EEG, pupil, temperature, heart rate

## Abstract

The eye drives non-visual (NV) responses to light, including circadian resetting, pupillary reflex and alerting effects. Initially thought to depend on melanopsin-expressing retinal ganglion cells (ipRGCs), classical photopigments play a modulatory role in some of these responses. As most studies have investigated only a limited number of NV functions, generally under conditions of relatively high light levels and long duration of exposure, whether NV functions share similar irradiance sensitivities and response dynamics during light exposure is unknown. We addressed this issue using light exposure paradigms spectrally and spatially tuned to target mainly cones or ipRGCs, and by measuring longitudinally (50 min) several NV responses in 28 men. We demonstrate that the response dynamics of NV functions are faster than previously thought. We find that the brain, the heart, and thermoregulation are activated within 1 to 5 min of light exposure. Further, we show that NV functions do not share the same response sensitivities. While the half-maximum response is only ∼48 s for the tonic pupil diameter, it is ∼12 min for EEG gamma activity. Most NV responses seem to be saturated by low light levels, as low as 90 melanopic lux. Our results also reveal that it is possible to maintain optimal visual performance while modulating NV responses. Our findings have real-life implications. On one hand, light therapy paradigms should be re-evaluated with lower intensities and shorter durations, with the potential of improving patients’ compliance. On the other hand, the significant impact of low intensity and short duration light exposures on NV physiology should make us reconsider the potential health consequences of light exposure before bedtime, in particular on sleep and circadian physiology.

## Introduction

By processing light information via dedicated pathways, the mammalian retina can engage not only in vision but also in light-dependent non-visual (NV) responses such as melatonin suppression, pupillary constriction, increase in body temperature and heart rate, and modulation of cortical brain activity. The effects of light on these responses have been shown to depend on intensity (Cajochen et al., 2000; Zeitzer et al., 2000, 2005; Prayag et al., 2019), duration (Chang et al., 2012), timing (Khalsa et al., 2003), temporal pattern (Gronfier et al., 2004; Zeitzer et al., 2011; Najjar and Zeitzer, 2016), and spectral distribution of the light stimulus (Brainard et al., 2001; Thapan et al., 2001; Najjar et al., 2014).

A body of evidence in human studies, including in blind individuals lacking classical visual photoreceptors (Zaidi et al., 2007), show a short wavelength sensitivity of NV functions (Cajochen et al., 2005; Revell et al., 2005; Münch et al., 2006; Lockley et al., 2006; Vandewalle et al., 2007; Mure et al., 2009; Gooley et al., 2010, 2012), with a peak around 480 nm (Brainard et al., 2001; Thapan et al., 2001; Najjar et al., 2014), close to the peak sensitivity of ipRGCs (Berson et al., 2002; Dacey et al., 2005). While this argues in favor of a primary role of intrinsically photosensitive retinal ganglion cells (ipRGCs) in NV functions through projections to a wide range of central targets (Gooley et al., 2003; Hattar et al., 2006; Fernandez et al., 2016), classical photoreceptors (rods, cones) have also been shown to play a role in such light-dependent responses, both in rodents and humans (Dkhissi-Benyahya et al., 2007; Gamlin et al., 2007; Mure et al., 2009; Gooley et al., 2010; Lall et al., 2010; Keenan et al., 2016).

A common strategy used to investigate the contribution of the retinal photoreceptors in NV responses, both in animals and humans, has been to exploit differences in spectral sensitivity between visual and NV photoreception. In humans, integrated responses have been observed under monochromatic blue and green light (Cajochen et al., 2005; Münch et al., 2006; Lockley et al., 2006; Vandewalle et al., 2007; Gooley et al., 2010, 2012; Daneault et al., 2012; Rahman et al., 2014; Segal et al., 2016), corresponding to sensitivities of ipRGCs (480 nm) and the photopic system (555 nm), respectively. This approach has been successful in separating relatively rod/cone versus ipRGCs contributions in light-dependent responses. However, the use of monochromatic lights or bright white light, at high light intensities, relatively long durations, with full visual field exposure and measure of a single NV response provide a limited understanding of the response dynamics. In addition, most studies did not consider that photoreceptors show different spatial distributions in the retina. While the highest density of cones is in the fovea, ipRGCs and rods are totally absent from this area and are distributed over the remaining visual field (Dacey et al., 2005; Liao et al., 2016; Hannibal et al., 2017).

The present study was designed to take into account, not only the differences in spectral sensitivity between photoreceptors, but also their differences in response dynamics and spatial distribution over the retina. We hypothesized that NV responses are influenced simultaneously by the spectral content/intensity, duration and spatial distribution of light exposure. Using blue-enriched and red-enriched light exposures tuned to target relatively specifically ipRGCs and classical photoreceptors, and measuring several NV functions during light exposure, we aimed to clarify whether NV functions share a common response dynamic, intensity and duration response curves.

Our four main hypotheses were as follows:

(1) During a 50 min exposure, blue-enriched white lights will produce higher NV response levels in comparison to red-enriched white lights, due to its higher melanopic content (∼230 vs. ∼90 melanopic lux);
(2) Adding an intense, central, white light spot to the blue or red-enriched white light stimulus will increase NV response levels, due to higher photoreceptor stimulation, and that the largest eccentricity will lead to a higher response due to the larger retinal surface area stimulated;
(3) Given the fast response of photoreceptors to light, NV responses will respond very rapidly, within a few minutes;
(4) Non-visual responses possess specific response dynamics and can be described by specific duration-response models.

## Materials and Methods

### Participants

Twenty-eight healthy male volunteers (40.7 ± 8.1 years) were recruited via advertisements at the different Lyon Universities and laboratories. Volunteers filled out questionnaires about their general health, sleep quality (Pittsburg Sleep Quality Index Questionnaire, PSQI), and their sleep-wake behavior (Horne and Ostberg Chronotype Questionnaire). Inclusion criteria were good sleep quality (PSQI score ≤5), no extreme chronotypes (Horne and Ostberg score between 42 and 58), no shift work nor transmeridian travel during the past 3 months, and a stable sleep-wake cycle (social jet-lag ≤2 h). Subjects with no evidence of pathology, psychiatric and sleep disorders were scheduled for an examination of visual functions. Visual acuity (Landolt Ring Test), contrast vision (Functional Acuity Contrast Test) and color vision (Farnworth D-15) were evaluated in order to exclude volunteers with visual dysfunctions, particularly color blindness and color vision deficiencies. Volunteers with glasses or contact lenses were not excluded as long as they had satisfied all the visual tests. All experimental procedures were carried out in accordance with the principles of the Declaration of Helsinki, and the protocol was approved by the Institutional Review Board and the local Ethics Committee (Comité de Protection des Personnes, Lyon, France). All subjects gave written informed consent.

### Overall Study Design

All participants were instructed to limit alcoholic beverages (maximum of two glasses per day), and restricted caffeine-containing beverages to one per day in the morning, starting seven days prior to the in-laboratory session. In addition, participants were instructed to maintain a regular sleep-wake schedule (bedtimes and wake times within ±30 min of self-targeted times) for the seven days before the experimental sessions. Compliance was verified by self-reported online sleep log and by wrist actigraphy (Actisleep, Actitrac, United States). Experimental sessions were conducted at the Inserm Platform for Research in Chronobiology at the Edouard Herriot Hospital in Lyon. On experimental day, participants’ arrival time was scheduled at 17:30. They were explained the protocol and prepared for the experiment under dim white-light condition of <5 lux. The average temperature of the room during the experimental sessions was 22.8 ± 0.3°C (mean ± sd).

#### Protocol and Light Exposure

**FIGURE 1:**
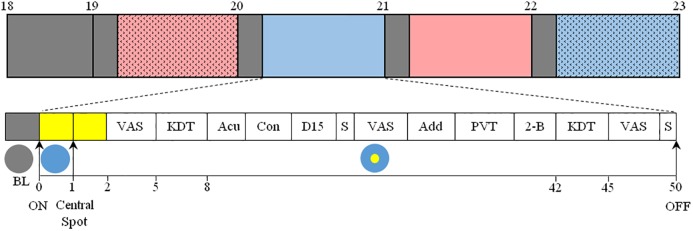
Top: Overview of the protocol. After arrival at 17:30, participants were maintained in dim light conditions (<5 lux, gray) until the beginning of the 4 sequential light pulses (BE-C1, blue; RE-C1, red; BE-C2, filled blue; RE-C2, filled red). The order of light conditions was balanced according to an orthogonal Latin square (see section “Materials and Methods”). Experimental sessions began at 19:00 and ended at 23:00. Each light condition consisted of 10 min in dim light (gray boxes, <5 lux) followed by 50 min of light. Bottom: Detail of each light pulse. After 10 min in dim light (gray box), light was turned on and the entire visual field was exposed (illustrated with a blue circle) to either blue- (BE) or red-enriched (RE) white light. This light was maintained until the end of the 50-min session. One minute after the initiation of the light exposure, a white light spot (illustrated with a yellow circle) was added in the central visual field, and was maintained until the end of the session. This central light spot occupied a field size of either 36° (C1) or 120° (C2). The measurements conducted are shown as: VAS, Visual Analog Scale; KDT, Karolinska Drowsiness Test; Acu, Acuity test; Con, Contrast sensitivity test; D-15, Farnsworth D-15 (color vision); S, Saliva collection; Add, Addition test; PVT, Psychomotor-Vigilance Task; 2-B, 2-back test. EEG, ECG, temperature and pupil diameter were recorded continuously between 19:00 and 23:00.

Prior to light exposure at 19:00, participants were seated in a comfortable chair, with their head placed in a head rest, in front of a table. Subjects’ eyes were located 58 cm above, and at an angle of 21° from, the gazing point on the table. The light source was overhead, placed 123 cm vertically above the gazing point and light was projected onto a horizontal table. White linen was used to mask other equipment in the test room and set the walls and floor to a homogeneous reflection factor. Subjects were exposed to four randomized 50-min light pulses between 19:00 and 23:00 with 10 min rest in dim white light conditions between each pulse (Figure 1). Within each light pulse, a blue-enriched white light (BE) or a red-enriched white light (RE) spectrum was switched on at 19:10. The BE or RE lights were used to stimulate ipRGCs differentially throughout the 50-min light exposure. The melanopic lux content was ∼230 for BE, compared to ∼90 melanopic lux for RE. After a first minute of full field light exposure to either BE or RE spectrum, a central white light spot was additionally turned on, and both lights remained on until the end of the pulse (min 50). The central light spot formed a white light circle centered around the fixation point on the table, of either 36 cm (corresponding to a field size of ∼36° in diameter, or ∼18° retinal eccentricity, C1), or 135 cm (corresponding to a field size of 120° in diameter, or ∼60° retinal eccentricity, C2).

The order of the four light conditions (BE-C1, RE-C1, BE-C2, RE-C2) across the 28 subjects was balanced according to an orthogonal Latin square such that each light condition was presented seven times as the first pulse (19:00–20:00), seven times as the second pulse (20:00–21:00) and so on. During light exposures, subjects were required to keep their eyes fixed on the gazing point. Compliance was verified via the video output of the eye tracker. Spectral and spatial characteristics of the lights used are shown in Supplementary Figures 1–3. Light spectra were calibrated to provide the following corneal photon flux to participants: 1.45 × 10^14^ photons/cm^2^/s for BE and RE, 5 × 10^14^ photons/cm^2^/s for BE-C1 and RE-C1, and 7.2 × 10^14^ photons/cm^2^/s for BE-C2 and RE-C2. Photopic, melanopic and other alpha-opic illuminances (Lucas et al., 2014) are given in Supplementary Table 1. Light was measured at 37 different locations in space with a spectroradiometer (JETI, Jena, Germany) in order to determine illuminances and spatial distribution of light exposure. The spectroradiometer was placed at the eye level of participants in the experimental situation.

### Measures

#### Karolinska Drowsiness Test (KDT)

In order to ensure artifact-free electroencephalography (EEG), electrocardiography (ECG), temperature and pupil diameter recordings, participants were asked to complete three Karolinska Drowsiness Test (KDT, 3 min each) procedures over the 50-min light pulses: (1) starting one minute prior to lights on (min -1 to 0, baseline in dim light, BL) and ending one minute after the addition of the central spot (min 1 to min 2), (2) from min 5–8 (KDT5), and (3) from min 42–45 (KDT42). During these episodes, participants were instructed to relax, fixate the gazing point on the table, and avoid blinking. For KDT5 and KDT42, the first minute was with open eyes, and the last two minutes with eyes closed. In this paper, we report data only from the eyes open segments. Overall, 5 eyes-open 1-min KDT segments were analyzed: BL, 1st min, 2nd min, 5th min, and 42nd min.

#### EEG

Continuous EEG signals were recorded using the Vitaport digital ambulatory system (Vitaport-4 digital recorder, TEMEC Instruments, Kerkrade, Netherlands) from silver/chloride electrodes placed at Fz (frontal), Cz (central), Pz (parietal), and Oz (occipital) scalp sites (10–20 international system). Active electrodes were referenced to the linked A1–A2 mastoids. Signals were filtered offline before spectral analysis, using a high pass filter at 0.3 Hz, a low pass filter at 45 Hz, and a notch filter at 50 Hz. All channels were recorded and stored at 256 Hz. All impedances were kept below 5 kOhm. A diagonal electrooculogram (EOG) derivation was also recorded to detect and remove artifacts on the EEG due to eye movements. Data from the Cz channel were analyzed and reported here.

EEG signals from the five 1-min segments derived from the KDT episodes were visually inspected and artifacts were removed. Across the four light conditions, an average of 509 ± 41 s (mean ± sd) was considered as artifacts and removed. This number was consistent across light condition: 450 s for BE-C1, 544 s for BE-C2, 528 s for RE-C1, 512 s for RE-C2. The total artifacts removed was 6.1% of the total EEG time segments analyzed (560 min). Artifact-free 2 s epochs were subjected to offline spectral analysis using Fast Fourier Transform (Prana, PhiTools, Strasbourg, France). Spectral resolution was 0.5 Hz, and a Hanning window was used. EEG absolute power was quantified between 0.5 and 45 Hz, and subdivided into the following bands: delta1 (0.5–2 Hz), delta2 (2.5–4 Hz), delta (0.5–4 Hz), theta1 (4.5–6 Hz), theta2 (6.5–8 Hz), theta (4.5–8 Hz), alpha1 (8.5–10.5 Hz), alpha2 (11–13 Hz), beta1 (13.5–25 Hz), beta2 (25.5–32 Hz), beta (13.5–32 Hz) gamma (32.5–45 Hz), slow-wave activity (1–7 Hz), 0.5–5.5 Hz, 9.5–10.5 Hz. Results from delta, theta, alpha2 (high alpha), beta and gamma are shown here.

#### Pupil Diameter

Continuous pupillary diameter was measured for both eyes using an infrared video pupil tracking system and software (ViewPoint Eye Tracker^®^, Arrington Research Inc., Mesa, Arizona) at a sampling rate of 30 Hz. Pupil diameter was analyzed with an algorithm developed in our laboratory (Matlab, 2015a, MathWorks Inc., Natick, MA, United States) for artifact rejection and data smoothing. Specifically, upper and lower threshold pupil diameter values were defined within normal physiological values. A circularity threshold of 0.7 was also applied. Values departing from the upper/lower and circularity thresholds were excluded. Data were smoothed using a locally weighted non-parametric regression fitting procedure (*loess*). Baseline diameter was calculated as the median of the pupil diameter measured during minute prior to lights-on (BL). Pupillary constriction was then calculated as the relative change from the baseline. Phasic pupil diameters were derived from the maximal pupil constriction measured during the first 5 s after the two light transitions: (1) dim light to full visual field exposure (1st min), and (2) addition of the central spot (2nd min). Tonic responses were calculated as the median pupil diameter during the last 10 s of min 1, min 2, min 5, and min 42. An example of pupillary constriction during the 50-min light exposure and the timing of measurements are shown in Supplementary Figure 5.

#### Temperature

Skin temperatures were collected continuously throughout the light sessions using skin thermocouples (iButtons, Dallas SemiConductors, United States) located in three places (wrist, ankle, and clavicle). Data were stored at 10 s interval. Analysis was carried out on median temperature values over the five 1-min segments derived from the KDTs. Subsequently, the distal-proximal gradient (DPG, wrist temperature-clavicular temperature) was calculated.

#### ECG

Two electrocardiogram leads were positioned on the sternum and the lateral thorax. The signal was recorded at 256 Hz on a Vitaport-4 digital system (TEMEC Instruments B.V., Kerkrade, Netherlands). Respiration measures were not collected. Across the four light conditions, an average of 37 ± 12 s (mean ± sd) was considered as artifacts and removed. This number was consistent across light condition: 53 s for BE-C1, 41 s for BE-C2, 25 s for RE-C1, 30 s for RE-C2. The total artifacts removed was 0.44% of the total ECG time segments analyzed (560 min). Heart rate variability analysis (HRV) of the ECG was conducted on artifact-free 30 s epochs to extract a measure of heart rate (HR) and of sympathovagal balance during light exposure (Prana, PhiTools, Strasbourg, France). Analysis was conducted on the five 1-min segments derived from the KDTs. Bands analyzed were: very low frequencies (VLF, 0–0.04 Hz), low frequencies (LF, 0.04–0.15 Hz) and high frequencies (HF, 0.15–0.5 Hz). The ratio LF/HF was used as a measure of sympathovagal balance (Otzenberger et al., 1998).

#### Salivary Melatonin

Saliva was collected 10 min before the start of the first light exposure session, in dim light conditions (<5 lux), and 13 min after the start of each light stimulus, and at the end of each light condition (min 49). The Buhlmann ELISA kit (Bühlman Laboratories, Allschwil, Switzerland), was used to determine melatonin concentrations. Limits of quantification for ELISA assay were 1.6–20.5 pg/ml. Intra-assay coefficients of variation (CVs) were 17.1% below 6 pg/ml and 8.1% for concentrations above. Inter-assay CVs were 23.3% below 6 pg/ml and 22.3% for concentrations above.

#### Cognitive Performance and Neurobehavioral Evaluation

Cognitive test performance was evaluated 25 minutes after lights-on via an Addition test, a Psychomotor Vigilance Test (PVT) and a 2-back test (see Figure 1). Each test generated a unique score per light condition which was subsequently analyzed. The addition test consisted of adding pairs of 2-digit numbers during five minutes. Correct answers were analyzed. After the addition test, participants completed a 10-min PVT. They were required to press a response button as fast as possible in response to the appearance of a visual stimulus on a computer screen which was presented randomly at intervals ranging from 3 to 7 s. Their median and slowest 10% reaction times were analyzed. In the 2-back test, participants were presented with letters. They were requested to determine whether or not the current letter was identical to the one presented 2 letters earlier. The presentation time for each letter was 1500 ms, inter-stimulus interval was 0 ms and time allowed for response was 1500 ms. The score analyzed for the 2-back test was the percentage of correct answers during a three-minute episode. All tests were programmed and presented on a small computer screen (22.4 × 12.6 cm) placed in the center of the visual field, on the fixation point.

#### Visual Performance

Visual performance was evaluated using the Landolt Ring Test for visual acuity, the Farnsworth D15 for color vision and the contrast sensitivity was determined with the functional acuity contrast test (F.A.C.T.). They were presented sequentially starting 8 min after lights-on (see Figure 1). The visual acuity score was determined with the Landolt Ring test. The side of the gap (left, right, up, or down) in the letter C had to be determined as the C-optotype becomes progressively smaller. The acuity score was recorded when participants made the first error. In the D-15 color arrangement vision test, participants had to arrange 15 colored disks in the correct color-coded order, forming a sequence of gradually changing hues. Misplaced disks resulted in deviated scores. The F.A.C.T. consisted of presenting a series of sine-wave gratings over five rows of different spatial frequencies (1.5 [A], 3 [B], 6 [C], 12 [D], 18 [E] cycles per degree). Each spatial frequency had 9 grating patches. The contrast step between each patch was 0.15 log units. Participants determined their last discernible level of the grating pattern for each of the five spatial frequencies (A, B, C, D, E) and a contrast sensitivity score was recorded.

#### Visual Analog Scores

Participants were asked to evaluate their subjective levels of alertness, stress level, visual acuity and mood via computerized 100 mm visual analog scales (VAS). The tests were presented sequentially at the beginning (min 3–5), in the middle (min 23–24), and at the end (min 46–48) of each 50-min light pulse. Data will be published elsewhere.

#### Duration Response Curves

To construct the percentage change duration response curves (DRC), response change (%) from baseline dim-light were plotted against time at min 0, 1, 2, 5, and 42. For the relative sensitivity DRCs, response change (%) obtained at min 0, 1, 2, 5, and 42 were normalized to their maximum at min 42 and plotted against time. A 4-parameter logistic model was then fitted in order to characterize the relationship between response and duration for each NV function. The equation for the model is shown below.

$$f(x)=d+\frac{a-d}{1+\left({}_{\bar{b}}^{x(-c)}\right)}$$

In the equation, *a* is the maximum response, *b* is the duration at which 50% of the maximal response is achieved (EC50), *d* is the minimum response and was constrained to zero, and *c* is a measure of the steepness of the rising portion of the curve. Curves were fitted using non-linear least-squares analysis in R (v. 3.2.1). As an indication of the goodness of the fits, the square of the correlation coefficient (*R*^2^) was calculated. Parameter estimates and *R*^2^ values for the DRCs are given in Supplementary Table 5.

### Statistical Analysis

**FIGURE 2:**
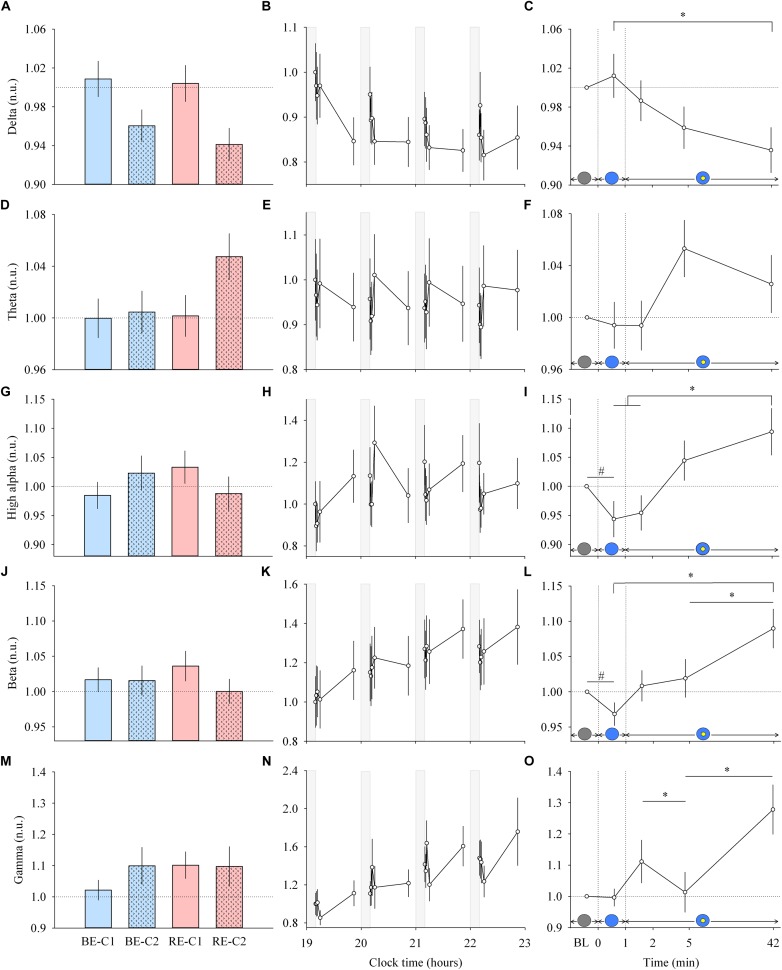
EEG response and dynamics during light exposure. (**A,D,G,J,M**; left column) Effect of light condition on each EEG band. EEG delta, theta, high alpha, beta and gamma absolute power densities are expressed relative to their respective dim light (min 0) values, in normalized units (n.u.). (**B,E,H,K,N**; middle column) Variation of EEG delta, theta, high alpha, beta, gamma during the protocol. Each pulse, corresponding to the average over the four light conditions, is shown in plain lines. Absolute EEG power densities for each band are expressed relative to their dim light level at 19:10, in normalized units (n.u.). Gray bars show the 10-min dim-light episode before each light exposure. (**C,F,I,L,O**; right column) Dynamics of the EEG during the 50-min light exposure. Absolute EEG power densities are expressed relative to their respective dim light (min 0) values, as normalized units (n.u.). Schematic representation of the light stimulus is shown above the time (min) axis. The gray circle indicates dim light. The blue- or red-enriched white light condition is illustrated by the blue circle. The yellow circle inside the blue circle represents the presence of the central light spot (C1 or C2) in addition to the full field exposure. The two vertical dotted lines indicate the light transitions, from dim light to blue- or red-enriched white light, and to addition of C1 or C2. Values represent the mean ± s.e.m. Dotted horizontal lines indicate the baseline dim-light level. #Significant pairwise comparison between min 0 and min 1. ^∗^Significant pairwise comparisons between min 1–42 time-points. *P*-values of all *post hoc* pairwise time comparisons are given in Supplementary Table 2.

**FIGURE 3:**
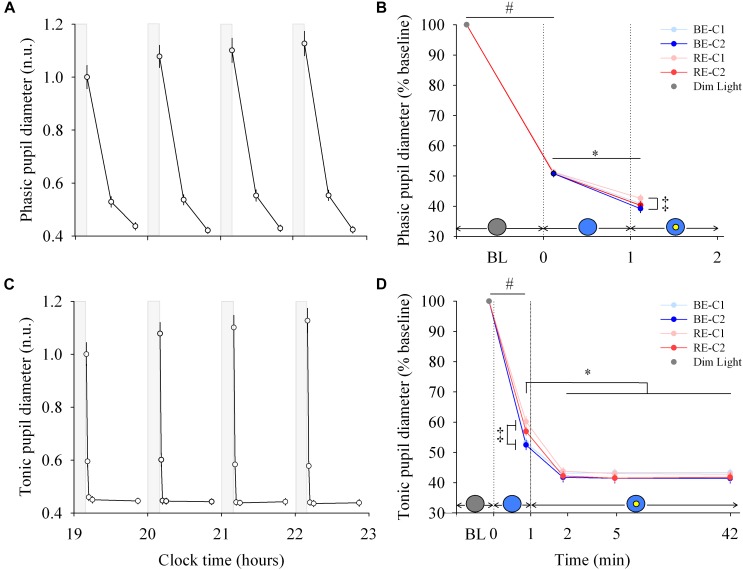
Pupillary response and dynamics during light exposure. **(A,C)** Variation of the phasic and tonic pupil diameter during the protocol. Each pulse, corresponding to the average over the four light conditions, is shown in plain lines. Pupil diameters are expressed relative to their dim light level at 19:10, in normalized units (n.u.). Gray bars show the 10-min dim-light episode before each light exposure. Note for the phasic diameter, the clock time axis is extended over the first 2 min only, and the gray bar representing dim-light is not to scale. **(B,D)** Dynamics of the phasic and tonic pupil diameters. Pupil diameters during the four 50-min light conditions are expressed as a percentage of the baseline diameter (min 0). Schematic representation of the light stimulus is shown above the time (min) axis. The gray circle indicates dim light. The blue- or red-enriched white light condition is illustrated by the blue circle. The yellow circle inside the blue circle represents the presence of the central light spot (C1 or C2) in addition to the full field exposure. The two vertical dotted lines indicate the light transitions, from dim light to blue- or red-enriched white light, and to addition of C1 or C2. Values represent the mean ± s.e.m. ^‡^Significant pairwise comparisons between light conditions. #Significant pairwise comparison between min 0 and min 1. ^∗^Significant pairwise comparisons between min 1–42 time-points.

**FIGURE 4:**
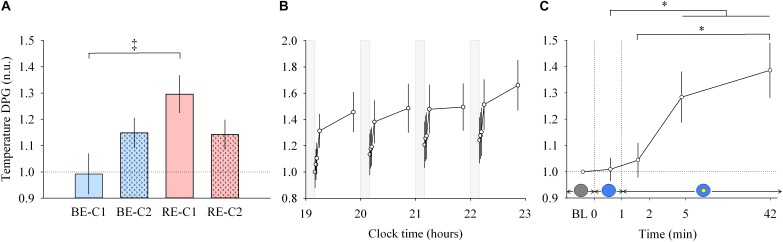
Distal to proximal skin temperature gradient (DPG) response and dynamics during light exposure. (**A**; left) Effect of light condition on the DPG. DPG absolute values are expressed relative to dim light (min 0) values, in normalized units (n.u.). (**B**; middle) Variation of DPG during the protocol. Each pulse, corresponding to the average over the four light conditions, is shown in plain lines. DPG absolute values are expressed as a change from their dim light level at 19:10, in normalized units (n.u.). Gray bars show the 10-min dim-light episode before each light exposure. (**C**; right) Dynamics of the DPG during the 50-min light exposure. DPG absolute values are expressed relative to dim light (min 0) values, in normalized units (n.u.). Schematic representation of the light stimulus is shown above the time (min) axis (right column). The gray circle indicates dim light. The blue- or red-enriched white light condition is illustrated by the blue circle. The yellow circle inside the blue circle represents the presence of the central light spot (C1 or C2) in addition to the full field exposure. The two vertical dotted lines indicate the light transitions, from dim light to blue- or red-enriched white light, and to addition of C1 or C2. Values represent the mean ± s.e.m. Dotted horizontal lines indicate the baseline dim-light level. ^‡^Significant pairwise comparison between light conditions. ^∗^Significant pairwise comparisons between min 1–42 time-points. *P*-values of all *post hoc* pairwise time comparisons are given in Supplementary Table 3.

**FIGURE 5:**
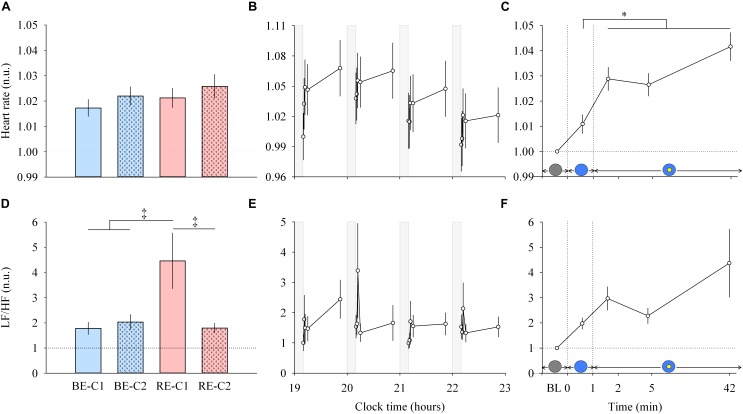
Heart rate and LF/HF ratio response and dynamics during light exposure. (**A,D**; left column) Effect of light condition on heart rate and LF/HF ratio. Absolute heart rate and the LF/HF ratio are expressed relative to their dim light (min 0) values, in normalized units (n.u.). (**B,E**; middle column) Variation of heart rate and LF/HF ratio during the protocol. Each pulse, corresponding to the average over the four light conditions, is shown in plain lines. Heart rate and LF/HF ratio absolute values are expressed as a change from their dim light level at 19:10, in normalized units (n.u.). Gray bars show the 10-min dim-light episode before each light exposure. (**C,F**; right column) Dynamics of the heart rate and LF/HF ratio during the 50-min light exposure. Absolute values are expressed relative to their respective dim light (min 0) values, as normalized units (n.u.). Schematic representation of the light stimulus is shown above the time (min) axis (right column). The gray circle indicates dim light. The blue- or red-enriched white light condition is illustrated by the blue circle. The yellow circle inside the blue circle represents the presence of the central light spot (C1 or C2) in addition to the full field exposure. The two vertical dotted lines indicate the light transitions, from dim light to blue- or red-enriched white light, and to addition of C1 or C2. Values represent the mean ± s.e.m. Dotted horizontal lines indicate the baseline dim-light level. ^‡^Significant pairwise comparisons between light conditions. ^∗^Significant pairwise comparisons between min 1–42 time-points. *P*-values of all *post hoc* pairwise time comparisons are given in Supplementary Table 4 for the heart rate and the LF/HF ratio.

Statistical analysis was performed in R v. 3.2.1 statistical environment^1^ using the *lme4*, *lsmeans*, and *car* packages. Given the two light transitions in our protocol, we analyzed the data in two steps. We compared the first minute of light exposure of the full visual field (min 1) to the dim-light baseline minute (min 0). In a second step, we compared time-points from min 1 to min 42. For min 0–1 analysis, absolute values were compared. For min 1–42 analysis, absolute values were expressed relative to baseline dim light values and compared. Each analysis for each response consisted of a Box-Cox transformation to normalize the data with the optimal power transform, followed by outlier detection (*outlierTest*, R). Overall, 17 outliers were removed out of a total 6528 sample points for all the NV response results described. Across subjects, six outliers were found and removed from the EEG (0.2%), five from the DPG (1.0%), two from the heart rate (0.35%), three from melatonin (1.3%), and one from cognitive performance tests (0.3%). No outliers were found for the phasic and tonic pupil diameters, visual performance tests and LF/HF ratio power-transformed datasets. For each response variable, we implemented a linear mixed-effects model to analyze the effects of factors “light,” “time,” and “order.” “Light” was the light condition (BE-C1, RE-C1, BE-C2, RE-C2); “time” was the duration within light exposure [5 time-points, before (BL) and during the 50-min light exposure (1st min, 2nd min, 5th min, 42nd min)]; and “order” was the presentation order of the light pulses (1, 2, 3, 4). Subject was a random factor in our model. Possible evidence of first-order autoregressive process was evaluated on the best-fitting models for the all the responses measured via the Durbin–Watson test. Resulting *p*-values were >0.05, excluding first-order autocorrelation in the all results observed except for the EEG theta (min 1–42), HR (min 0–1), LF/HF (min 1–42), and DPG (min 0–1). Given that there was no effect of time for those four response segments, we exclude the risk of a false-positive time effect due to a first-order autoregressive process. In all linear mixed model analyses, a likelihood ratio test (LRT) was performed to test the significance of the model (set at *P* < 0.05). The full model was evaluated first, and if not significant, factors were dropped. Conditional on a significant main effect of the factors above and/or interaction in our model, pairwise contrasts were inspected using least square means, with the *p*-value adjusted for multiple comparisons (Tukey method). These *post hoc* pairwise comparisons were carried out with *lsmeans* package. Plots (Figure 2–5) show the absolute values of each response relative to its dim-light values, in normalized units (n.u.). Variation during the 4 h of the protocol, as a change from the 19:10 dim light level, is also shown for the EEG, pupil diameter, DPG and heart rate variability.

## Results

### Cortical EEG Is Influenced Within 1 Min of Light Exposure

Each light pulse induced a systematic effect on most of the EEG bands that disappeared in the short 10-min interpulse interval (dim light) during the 4 h protocol (Figure 2B,E,H,K,N; middle column). Statistical analysis of the EEG activity did not show a main effect of light condition (Figure 2A,D,G,J,M; left column), nor an interaction “light” × “time” for both min 0–1 and min 1–42 segments. Within light exposures, for min 0–1, a main effect of time was obtained for high alpha (χ^2^ = 10.87, df = 1, *P* = 0.00098) and beta (χ^2^ = 5.21, df = 1, *P* = 0.022). *Post hoc* comparison revealed that the EEG activity showed strikingly rapid responses, with a significant decrease in high alpha and beta activities within the first minute of light exposure to BE or RE conditions (Supplementary Table 2).

From min 1–42, a main effect of time (Figure 2C,F,I,L,O; right column) was also found for most EEG bands (delta, χ^2^ = 10.63, df = 3, *P* = 0.014; theta, χ^2^ = 6.21, df = 3, *P* = 0.10, high alpha, χ^2^ = 14.1, df = 3, *P* = 0.0028; beta, χ^2^ = 14.04, df = 3, *P* = 0.0029; gamma, χ^2^ = 14.28, df = 3, *P* = 0.0025). *Post hoc* examination revealed important differences in the dynamics of cortical EEG (Supplementary Table 2). Delta activity continued to decrease up to the 42nd min of light exposure. This was different for fast EEG bands, where a bimodal response was observed. High alpha and beta activities increased over time after an initial rapid decrease. The addition of C1 or C2 light exposure did not yield any rapid change in EEG activity. There was also no difference in response levels between the two central field sizes (C1 and C2).

### Phasic and Tonic Pupil Diameters Are Differentially Modulated by Light Exposure

Each light pulse induced a systematic effect on both the phasic and tonic diameters that disappeared in the short 10-min dim light interpulse interval (Figure 3A,C; left column). For the phasic pupil diameter response between min 0–1, a main effect of time was observed (χ^2^ = 616.5, df = 1, *P* < 2.2E-16), but no effect of light condition or interaction of “light” × “time.” There was a main effect of “light” (χ^2^ = 12.30, df = 3, *P* = 0.006) and “time” (χ^2^ = 296.4, df = 1, *P* < 2.2E-16) for the phasic pupil diameter for the min 1–2 segment, with no significant interaction of “light” × “time.” Figure 3B (right column, upper panel) shows the phasic pupil diameter assessed after the two light transitions (when the full visual field was exposed, and when the central spot was subsequently added, respectively. See Supplementary Figure 5 for details). When light was initially switched on, the phasic constriction reached ∼49% (sd = 5.4), regardless the light condition (*P* < 0.0001). When the central light was turned on (at the end of min 1), phasic constriction was further increased by ∼10% (*P* < 0.0001) on average, and was higher under BE-C2 light compared to RE-C1 light (*P* = 0.0052). Phasic diameter did not differ between the two field sizes (C1 and C2).

For the tonic diameter between min 0–1, the interaction between “light” and “time” was significant (χ^2^ = 24.57, df = 3, *P* = 1.90E-5). From min 1–42, a significant interaction of “light” × “time” was obtained (χ^2^ = 38.17, df = 3, *P* = 1.62E-5). Figure 3D (right column, lower panel) shows the tonic constriction evaluated at 5 time-windows (BL, 1st, 2nd, 5th, 42nd min). When light was turned on, the tonic constriction was ∼44% (sd = 8.6) on average (*P* < 0.0001). Tonic constriction was greater with BE compared to RE light exposure (47%, sd = 8.1 vs. 41%, sd = 8.1), respectively, (*P* < 0.0002). Adding the central light increased tonic constriction by 10–16% (*P* < 0.0001, mean = ∼57%, sd = 7.5) and pupil constriction stayed at this level until the end of the light exposures for all light conditions. There was no difference in constriction levels between the two central field sizes (C1 and C2).

### Body Temperature (DPG) Increases Within 5 Min of Light Exposure and Continues to Increase Up to 42 Min

Similar to other responses measured, light pulses induced a systematic effect with a return to baseline levels in the short 10-min dim light interpulse interval (Figure 4B, middle). Between min 0–1, no main effect of “light” or “time” and no effect of the interaction between “light” and “time” were detected. For min 1 to 42, there was no interaction “light” × “time” but a main effect of “light” (χ^2^ = 13.92, df = 3, *P* = 0.00016; Figure 4A, left) and “time” were found (χ^2^ = 13.92, df = 3, *P* = 0.0030; Figure 4C, right). *Post hoc* analysis revealed that the RE-C1 condition was higher than BE-C1. As for the DPG increase over time, it was significant after only 5 min of light exposure (*P* = 0.030), and further increased after 42 min of light exposure (*P* = 0.0009) compared to min 1 levels. There was no difference between the two central field sizes (C1 and C2). Note that the ∼1°C (0.93°C) change in the magnitude of the DPG, induced by only 50 min of light exposure, is approximately one and half times (factor 1.48) the amplitude of the circadian component of the DPG rhythm over the 24 h (Kräuchi and Wirz-Justice, 1994). Thus, the light drive appears to be approximately 43 times stronger than the circadian drive on a per minute basis.

### Light Increases Heart Rate Within 2 Min and Continues to Influence the Cardiovascular System Over 42 Min

Light pulses induced a systematic effect on the heart rate (Figure 5B, middle column, upper panel), with a return to baseline levels in the 10-min interpulse interval (dim light). From min 0 to min 1, there was no main effects of “light” or “time”, and no effect of the interaction “light” × “time” for the heart rate. From min 1–42, there was no difference between the four light conditions (Figure 5A, left column, upper panel), and no “light” × “time” interaction. A main effect of time was observed (χ^2^ = 27.62, df = 3, *P* = 4.38E-6; Figure 5C, right column, upper panel). *Post hoc* tests revealed that this increase in heart rate was significant after the addition of the central spot (C1 or C2), at min 2. Specifically, this increase was significant compared to the previous minute (*P* = 0.0098) when the entire visual field was only exposed to red-enriched or blue-enriched white light. Heart rate continued to increase significantly at min 5 (*P* = 0.031) until the 42nd min of light exposure (*P* < 0.0001). No difference was observed between the two central field sizes (C1 and C2).

For heart rate variability (LF/HF ratio) between min 0–1 segment, an interaction of “light” × “time” was obtained (χ^2^ = 9.178, df = 3, *P* = 0.027). For min 1 to min 42, no interaction of “light” × “time” was found. A main effect of “light” was detected (χ^2^ = 39.57, df = 3, *P* = 1.32E-8; Figure 5D, left column, lower panel). *Post hoc* tests found that the LF/HF ratio was significantly higher during RE-C1 light exposure compared to RE-C2 (*P* = 0.0001), BE-C1 (*P* < 0.0001) and BE-C2 (*P* = 0.0001). A main effect of time was not observed (χ^2^ = 4.84, df = 3, *P* = 0.18; Figure 5F, right column, lower panel). Although not statistically significant, the LF/HF ratio, an index of the sympathovagal balance, showed a time course similar to that of HR, with an increasing trend between baseline and the 42nd min. LF/HF response amplitudes did not differ between C1 and C2 field sizes.

### Each Non-visual Response Has a Unique Duration-Response Curve

**FIGURE 6:**
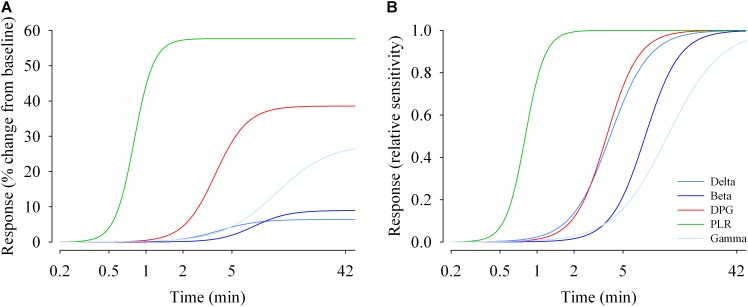
Duration response curves of non-visual functions in humans. The response magnitudes were plotted against duration of light exposure and fitted with a 4-parameter logistic model. **(A)** Response change (% change from baseline dim light) during light exposure differed between functions. The maximal responses vary between ∼6% (EEG delta activity) and ∼60% for the pupillary light reflex (PLR; tonic pupil response). **(B)** Relative responses reveal that sensitivity differs between non-visual functions. EC50s (half-maximum values) range between ∼48 s (tonic pupil response) and ∼12 min (EEG gamma activity). Parameter estimates and *R*^2^ values are given in Supplementary Table 5.

EEG (delta, beta and gamma activity), DPG and tonic pupil diameter data were fitted with a 4-parameter logistic function (Figure 6A, left). The *R*^2^ ranged from 0.80 (EEG gamma activity) to 1.0 (PLR tonic pupil diameter). Responses differ in their magnitude (maximum), and in their sensitivity (EC50). The half-maximum values (EC50) for EEG delta, beta and gamma activity were ∼3.9, 7.6, and 11.5 min, respectively. For the tonic pupil diameter and the DPG, EC50 was 0.8 and 3.7 min, respectively (see Supplementary Table 5). The 4-parameter logistic function on the normalized values is also shown (Figure 6B, right).

### Melatonin Change Within Each Light Exposure Was Similar in All Conditions

Change in melatonin levels within light exposure (from min 14 to min 48) was similar with all light pulses (Supplementary Figure 4). There was no interaction “light” × “time” nor a main effect of “light” or “time” on melatonin change. There was no significant increase of melatonin over time (19:00–23:00), implying that all conditions similarly suppressed melatonin secretion.

### Cognitive Performances Are Similar in All Light Conditions

**FIGURE 7:**
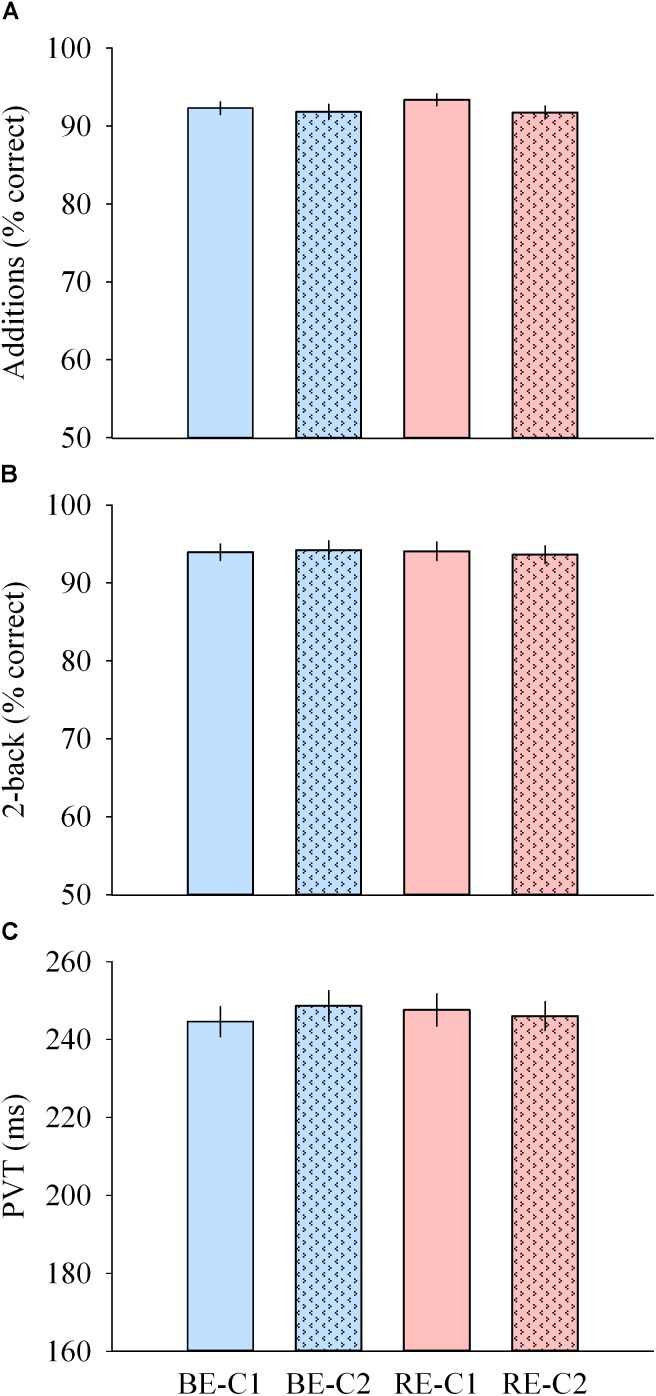
Cognitive performance during light exposure. **(A)** Number of correct additions (top), **(B)** percentage of correct 2-back answers (middle) and **(C)** median PVT (bottom) observed for the four light conditions. Values are mean ± s.e.m. No differences were observed between light conditions.

Performances to addition, median PVT (as well as the slowest 10% reaction times) and 2-back tests were not different across light pulses (Figure 7A–C).

### Visual Performance Is Not Affected by Addition of the Central Spot

**FIGURE 8:**
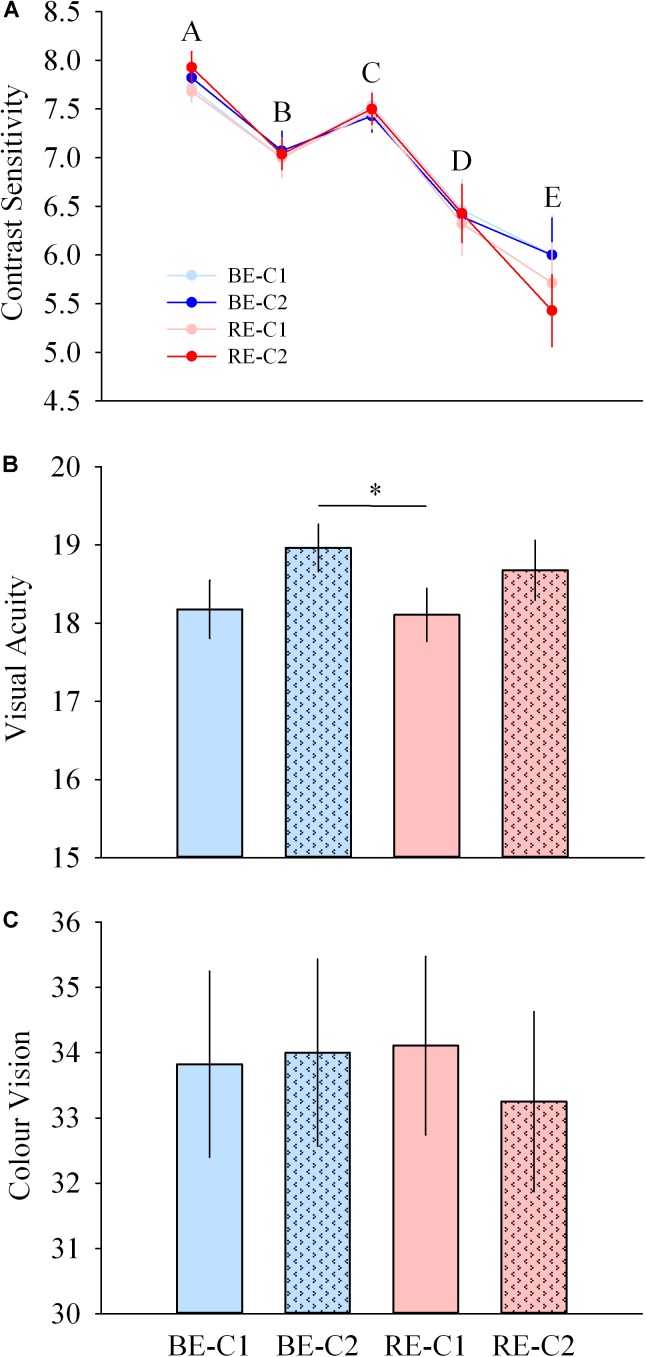
Visual performance during light exposure. **(A)** Contrast sensitivity at 5 spatial frequencies (top), **(B)** acuity (middle), and **(C)** color vision (Farnsworth D15, bottom) scores observed for the four light conditions. Values are mean ± s.e.m. ^∗^Significant difference between light conditions.

Contrast sensitivity was not different across light pulses, for all spatial frequencies. Acuity was higher with BE-C2 compared to RE-C1 (*P* = 0.040). Color vision was similar with all light pulses (Figure 8A–C).

## Discussion

Little is known about the sensitivity and response dynamics of NV functions in humans. Here, we reveal three characteristic features. Firstly, light influences some aspects of NV physiology much faster than previously thought. Secondly, each NV response is defined by a different duration function with specific saturating level, initiation level and response dynamics. Thirdly, the NV effects of light do not depend on the spatial distribution of the light stimuli used in our study.

### Light Influences Non-visual Physiology Much Faster Than Previously Thought

In terms of temporal response dynamics, studies have investigated NV responses to light at different time-scales, from a few milliseconds of light exposure (Zeitzer et al., 2011; Najjar and Zeitzer, 2016; Rahman et al., 2017) to several hours (Cajochen et al., 2000; Zeitzer et al., 2000, 2005; Lockley et al., 2006; Rahman et al., 2014). However, the dynamics of NV responses during exposure to a range of light intensities are unknown. Mure et al. (2009) showed that 5 min of 480 nm light exposure at 10^13^ photons/cm^2^/s (∼33 melanopic lux) sufficient to elicit phasic and tonic responses of the pupillary light reflex in humans. Heart rate has been shown to be increased by 10 min of continuous white light (100 lux, ∼50 melanopic lux, Scheer et al., 1999, 2004). Cajochen et al. (2005) showed that cardiac activation could even be sustained for 20 min following a 2 h light exposure (∼70 melanopic lux). Melatonin suppression has been observed for long durations (6.5 h) at relatively low intensity (EC50 ∼ 100 lux, Zeitzer et al., 2000). Using shorter durations, melatonin is suppressed by ∼20% with 12 min of light exposure at high intensity (9500 lux, 4100K, ∼6700 melanopic lux, Chang et al., 2012; Rahman et al., 2018). For cortical activity, light exposure has been shown to influence the EEG either following extended periods (2.5–6.5 h) of light exposure at low light intensity (12 melanopic lux, Phipps-Nelson et al., 2009; 72 melanopic lux, Rahman et al., 2014), or in a relatively shorter time (1–1.5 h) but with higher intensities (>3000 melanopic lux, Badia et al., 1991). For longer duration (6.5 h) of white light exposure, responses between 3 and 9100 lux (2–6000 melanopic lux) have been modeled and follow a logistic curve (Cajochen et al., 2000). Light exposure, concomitant with cognitive tasks, has been shown to modulate cortical activity after short durations. Vandewalle et al. (2010, 2011, 2013) showed that light impacts the cortex within tens of seconds. However, in those studies, light exposure was carried out simultaneously with a cognitive task execution, which in itself activates cortical regions. In the absence of simultaneous cognitive task, rapid transient effects have been shown on the fMRI within ∼15 s (Vandewalle et al., 2007), but in subcortical regions only. One study showed an effect of light on cortical activation (quantified by fMRI) in the absence of a simultaneous cognitive task (Hung et al., 2017).

While these studies describe the effect of light at different durations and intensities, they do not reveal the underlying response dynamics. In other words, how long does it take to elicit a NV response within a light stimulus? At the retinal level, light is captured and transduced at the photoreceptor level within milliseconds and induces electrophysiological responses within seconds and over a large range of light intensities. Our results here show that the pupil and the EEG power density can be modulated within 1 min of light exposure, at relatively low light intensities (∼90 melanopic lux), and that this effect does not diminish over time during light exposure (up to ∼50 min). The results also show that the cardiovascular system (HR and HRV), and the highly integrated thermoregulation system (DPG), can be modulated within 2 to 5 min, respectively. To the best of our knowledge, these results are the first to demonstrate that light can impact cortical activity, cardiac physiology, and temperature regulation in a strikingly rapid manner, at least much faster than previously thought.

Neuroanatomical substrates that can underpin this rapid effect of light are ipRGCs, the sole conduit of photic information to candidate regions that are implicated in alertness, heart rate, cognition, temperature, EEG activity, including the suprachiasmatic nucleus (SCN), the ventrolateral preoptic nucleus (VLPO), the locus coeruleus, the medial amygdala, the lateral habenula and the subparaventricular zone (Gooley et al., 2003; Hattar et al., 2006). The locus coeruleus in particular plays a central role in a number of physiological functions including arousal and autonomic activity (Samuels and Szabadi, 2008). Other candidates may be the hippocampus and the left thalamus which in humans have been shown to respond within 15 s to monochromatic 473 nm light exposures (Vandewalle et al., 2007), at intensity levels (∼80 melanopic lux) similar to those we used in our study (minimum of ∼90 melanopic lux). Direct input to such regions via ipRGCs, either *per se*, or via modulation by rods and/or cones, may therefore modify activity in these regions and be involved in these observations.

### Non-visual Functions Are Defined by Different Temporal Dynamics and Duration Response Curves

To our surprise, a main effect of the light condition was not observed in most responses. The four light conditions elicited similar responses for temperature (DPG), heart rate, EEG activity, cognitive performance, and melatonin suppression, in spite of their different intensities, ranging from ∼90 (RE) to ∼615 melanopic lux (BE-C2). We propose three possible explanations for this result: (i) the light intensities we used were below the responses thresholds, (ii) the range of 90–615 melanopic lux was insufficient to produce significant differences in responses, or (iii) the four light conditions were saturating.

(i) Since a main effect of time was observed for temperature, heart rate and EEG responses, we exclude the possibility that the light intensities we used were below response thresholds and that they did not impact temperature regulation, cardiovascular and cortical activities. We also found an average median reaction time (PVT) of ∼245 ms (sd = 20.49) in all four light exposures. In low light conditions, and at the same circadian phase, the reaction time (PVT) is ∼334 ms in constant routine conditions (Cajochen et al., 1999) and ≥ between 300 and 390 ms in forced desynchrony (Silva et al., 2010). Therefore, our light exposures did also impact psychomotor performance, suggesting that the light intensities we used were above the response thresholds.

(ii) We also exclude the possibility that the range of intensities used was not large enough. Using illuminances ranging from 3 to 9100 lux (2–6000 melanopic lux) to measure a range of NV responses (Cajochen et al., 2000; Zeitzer et al., 2000), EC50s were obtained with only 90–180 lux (60–120 melanopic lux) of light exposure. Thus, with a range spanning ∼525 melanopic lux in our study, it is legitimate to expect different magnitudes of responses between our light pulses.

(iii) Did we observe saturated responses? Saturation levels of a range of NV responses have been evaluated in separate studies for a 6.5 h white light exposure. For EEG theta-alpha (5–9 Hz) power, saturation of the response (90% of the maximal effect of light) was obtained at 180 lux (∼120 melanopic lux, Cajochen et al., 2000). In the same study, saturation levels were ∼170 lux (∼110 melanopic lux) and 390 lux (∼260 melanopic lux) for subjective alertness (KSS) and slow-eye movements, respectively. Using a fixed duration of light exposure (6.5 h), Zeitzer et al. (2000) found saturation of melatonin suppression at ∼200 lux. On the other hand, using a fixed intensity (>7500 lux, Chang et al., 2012), saturation of melatonin suppression is predicted to occur after 7.9 h of light exposure. Altogether, these results suggest that light levels of 90 melanopic lux as we used in our study should not saturate most responses, in particular for light pulses as short as 50 min in duration. However, our mathematical modeling (discussed further below) shows that all responses do reach a saturating response within 50 min of light exposure. Therefore, the reason we did not find differential effects between our four light conditions might be related, in fact, to a saturation of the responses over time.

In terms of dynamic responses, our mathematical modeling shows that NV functions do not share identical sensitivity curves, but that each NV function possesses (i) a unique sensitivity to light (Figure 6A,B), (ii) a specific range in response amplitude, constrained by light intensity and exposure duration (Figure 6A). Such non-overlapping of sensitivity has been shown for illuminance-response curves for pupil dilation and behavioral phase shift responses in the hamster (Hut et al., 2008). Here, we find that the maximum response amplitude vary between ∼6% (EEG delta activity) and ∼60% (tonic pupil diameter). We also reveal that EC50s range from 0.8 min (tonic pupil diameter) to 11.5 min (EEG gamma activity). That NV functions differ in terms of sensitivities is in accordance with findings showing that melatonin suppression and circadian phase shifting exhibit different intensity and duration sensitivity functions (Zeitzer et al., 2000, 2005; Chang et al., 2012), and that melatonin suppression cannot be used as a proxy of circadian phase shifting (Rahman et al., 2018).

### Non-visual Responses Do Not Necessarily Depend on Spatial Distribution of Light

In terms of spatial sensitivity, recent data on the spatial distribution of photoreceptors in the human retina demonstrate that ipRGCs are absent from the fovea (9° eccentricity) and are present relatively homogeneously throughout the peripheral retina (Liao et al., 2016; Hannibal et al., 2017). Only a few studies have investigated spatial stimulation of the retina. They showed that melatonin suppression in humans is stronger with illumination of the nasal retina compared to exposure of the temporal retina (Visser et al., 1999; Rüger et al., 2005a). Another study showed that exposure of the superior retina was more efficient in suppressing melatonin compared to inferior retinal stimulation (Glickman et al., 2003). These studies, while not accounting for the field size of the stimuli used, indicate that the retinal locus stimulated by light can alter the amplitude of NV responses. A relationship between field size and NV response level has been shown recently by Joyce et al. (2016) with the pupillary light reflex. Increasing foveated field size diameter from 10° to 20° increased post-illumination pupillary response but further increase to 30° and 40° did not produce any further change. Our results are in agreement with this finding as we did not find any differences between a 36° (C1) and 120° (C2) field size. Taking into account that ipRGCs are absent within 9° of eccentricity in humans (Liao et al., 2016), our results are in accordance with Joyce et al. (2016), and indicate that most of the response is driven by ipRGCs within the central 18° eccentricity.

A number of limitations of our study deserve to be mentioned. First, a relatively limited range of intensities were used in our study (range of 0.84 log melanopic lux). This might explain the absence of difference between light pulses for some responses. A larger range of intensities might have resulted in larger differences. Secondly, our study was carried out between 19:00 and 23:00, and it is possible that we would have obtained different responses at other times (Rüger et al., 2005b). Indeed, some NV responses have been shown to be dependent on time of day, due to combined influences of circadian phase and homeostatic processes (Münch et al., 2012). Third, our central white light spots (C1 and C2) did not specifically target cones or ipRGCs in terms of spatial distribution. We determine that the C1 light spot we used exposed ∼3.5% of the retinal surface area, and C2, ∼37.5%. Our calculations reveal that C1 covered 7% of ipRGCs and 16% of cones, and C2 covered ∼75% of ipRGCs and 81% of cones (Curcio et al., 1990; Liao et al., 2016). Therefore, the increased responses observed with C1/C2 addition could result either from an additional stimulation of cones, or an additional activation of ipRGCs, or both. Moreover, although our light conditions were balanced, the fact that only 10 minutes of dim light separated the different light conditions may have decreased differences between conditions. We also cannot exclude involvement of visual processing in our EEG responses. While the sigmoidal response dynamics observed over time is compatible with ongoing drive by NV pathways, the magnitude of the EEG responses may have been provided by a combination of both NV and visual processing during our KDT segments. This combined envelope and its disentanglement remain to be investigated. Finally, this study was carried out in men only, and while similar results might be obtained in women as well, further studies are needed to assess potential gender differences. These limitations, however, do not invalidate the relevance of the observed effects of light exposure and the conclusions of our study.

## Conclusion

We have demonstrated that polychromatic lights with different spectral content and spatial distribution stimulate several NV responses with different dynamics and sensitivities. Responses can be very rapid, within 1–5 min of light exposure (temperature, cortical EEG, pupil diameter, cardiac output) even at room light levels (90 melanopic lux). In addition, NV responses can be saturated by relatively low light levels, as low as 90 melanopic lux, for most responses (with the possible exception of the tonic pupil diameter). Our results provide insights on temporal integration of light-sensitive regions in the central nervous system. In rodents, all subtypes of ipRGCs respond within 30 s to light levels comparable to ours (Zhao et al., 2014), and their response does not dampen over long durations of light exposure (Wong, 2012). We reveal that, except the pupillary light reflex, NV responses continue to increase over 10-40 min of light exposure. This suggests that temporal integration occurs not only in the retina but also downstream in NV structures and takes several minutes before its effect can be fully observed in NV responses. Finally, we also show that it is possible to maintain optimal visual performance while modulating NV responses.

Our findings have real-life implications. On one hand, that light exposures can elicit strong NV responses at relatively low intensities and short durations, could therefore improve compliance to light therapy protocols by making them more practical and comfortable. Before we switch from “long duration bright light therapy” to “short duration room light therapy,” however, further investigations are warranted. We also could imagine devices or technologies targeted at modulating peripheral light exposure to enhance NV responses (e.g., vigilance, cognitive performance, mood, etc.) without negatively impacting visual performance. Finally, the high effectiveness of low intensity and short duration light exposures on NV responses adds to the body of evidence claiming the potential risks of light exposure before bedtime on sleep and circadian physiology (Cajochen et al., 2011; Chellappa et al., 2011; Santhi et al., 2012; Czeisler, 2013; Chang et al., 2015; Gringras et al., 2015; Hatori et al., 2017; Prayag et al., 2019).

## Data Availability

The raw data supporting the conclusions of this manuscript will be made available by the authors, without undue reservation, to any qualified researcher.

## Author Contributions

CG conceived the experiments. CG and DD designed the experiments. ASP, SJ, PA, DD, and CG collected the data. ASP conducted the data analyses for EEG, HR, temperature, cognition, vision, melatonin, and pupillary light reflex. SJ conducted the data analyses for pupillary light reflex. DD conducted the data analyses for light spectra and irradiances. ASP and CG interpreted the data and wrote the manuscript. SJ, PA, and DD provided edits.

## Conflict of Interest Statement

Over the past 2 years, CG has had the following commercial interests related to lighting: consulting contracts with Dayvia, Lucibel; unrestricted equipment gifts from Dayvia, HeLight. CG is listed as an inventor on 2 patents filed by Inserm Transfert on a wearable health and lifestyle device, and on a lighting apparatus with optimum stimulation of non-visual functions. The remaining authors declare that the research was conducted in the absence of any commercial or financial relationships that could be construed as a potential conflict of interest.

## Footnotes

1 http://cran.r-project.org
