# Supplementary material for: Dynamics of Non-visual Responses in Humans: As Fast as Lightning?
Source: Front Neurosci. 2019 Mar 5;13:126. doi: 10.3389/fnins.2019.00126 (PMC6411922; doi:10.3389/fnins.2019.00126)
Supplement: Supplementary file 1 [file Data_Sheet_1.pdf]

Supplementary Material

Dynamics of non-visual responses in humans: as fast as lightning?

Abhishek S. Prayag, Sophie Jost, Pascale Avouac, Dominique Dumortier, Claude Gronfier*

*** Correspondence:** Claude Gronfier: claude.gronfier@inserm.fr

1 Supplementary Figures

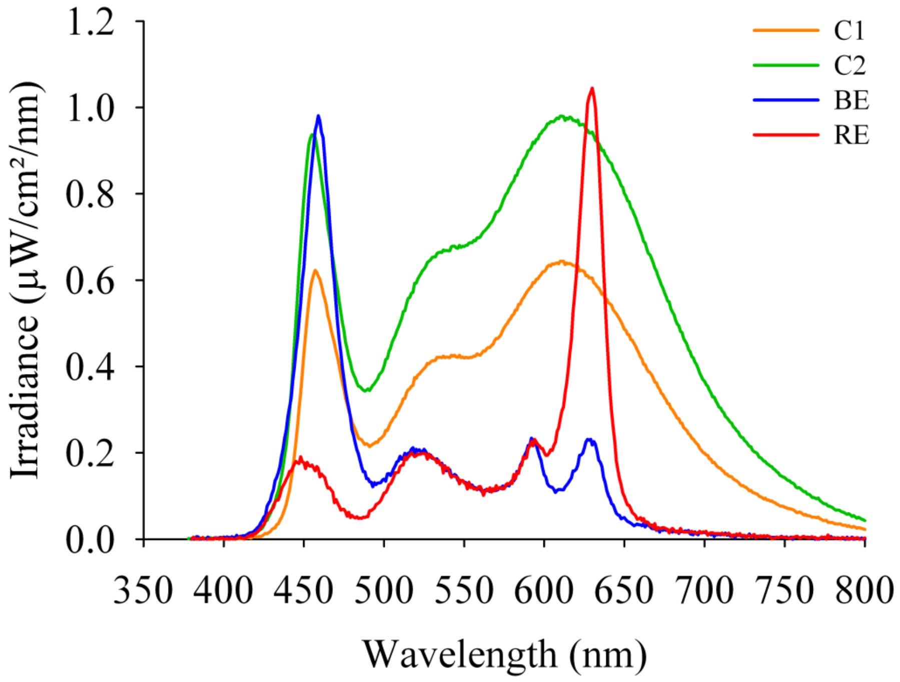

Supplementary Figure 1. Individual light spectra: blue-enriched (BE), red-enriched (RE), and central light (C1 and C2).

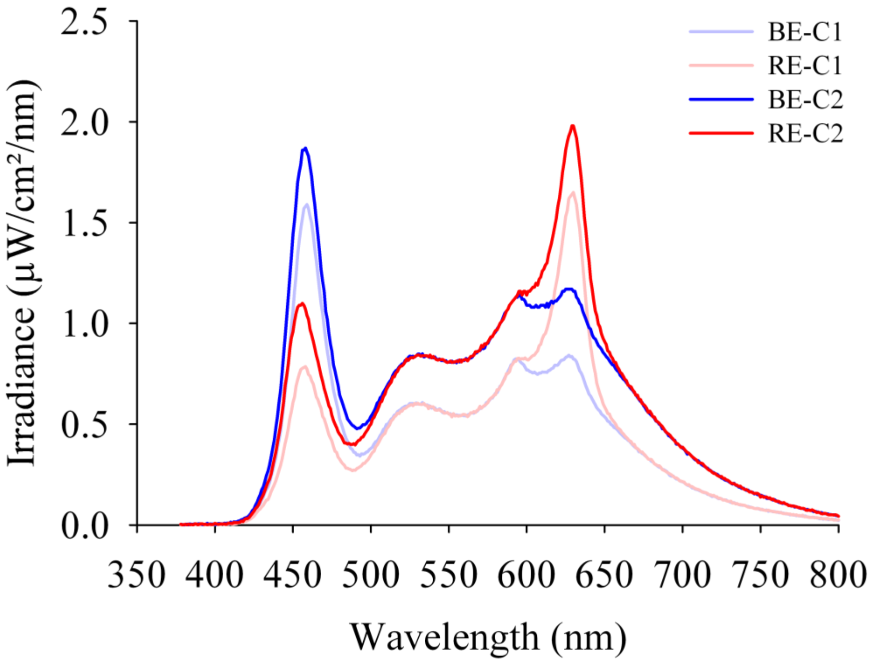

Supplementary Figure 2. Combined light spectra of BE or RE with C1 and C2.

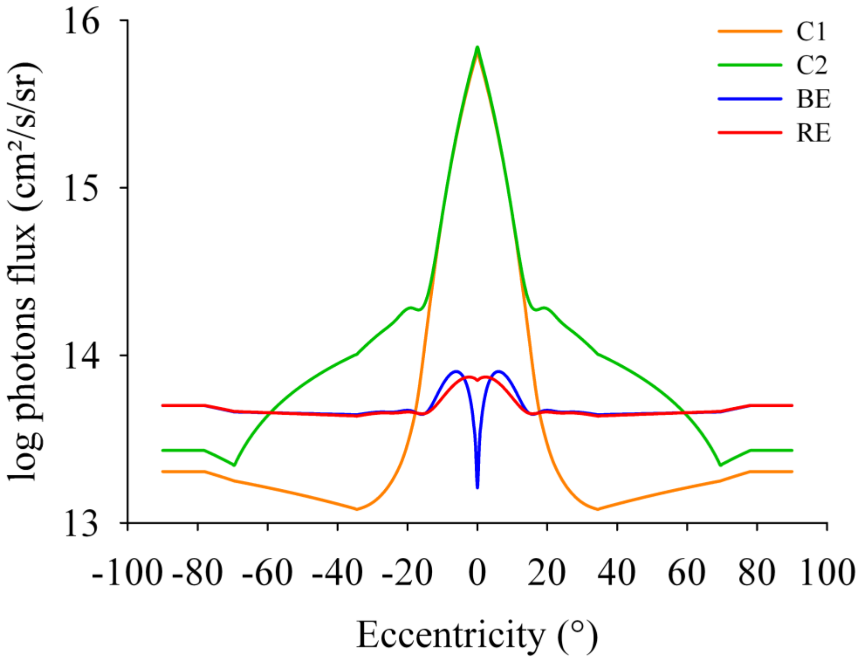

Supplementary Figure 3. Spatial distribution of light intensity as a function of eccentricity.

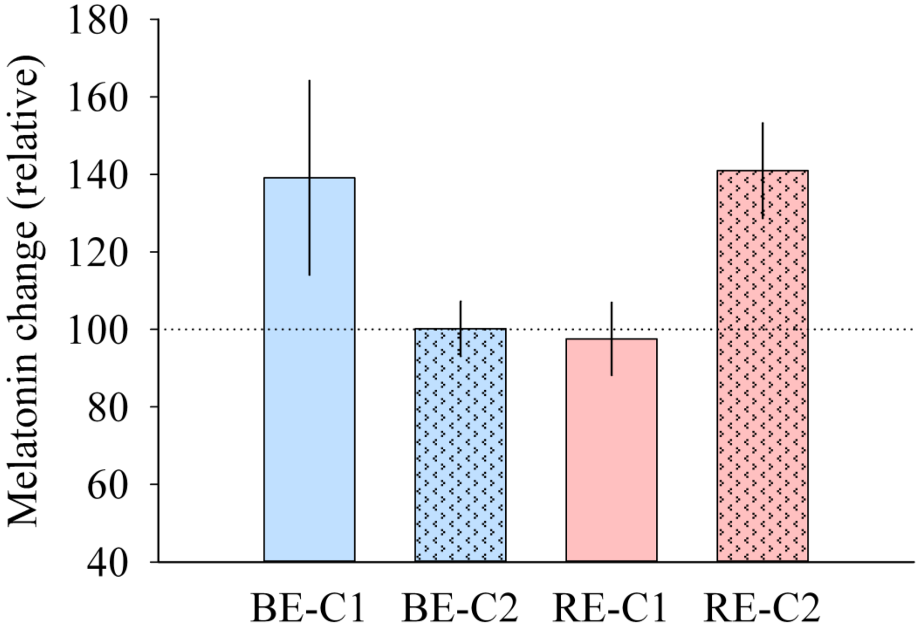

Supplementary Figure 4. Relative change of melatonin concentration within each light condition. Values represent the relative change of melatonin concentration (mean ± s.e.m) between min 14 and min 48. The dotted horizontal line indicates the relative level at min 14. An increase above this line indicates an increase in secretion of melatonin during the light pulse. No significant effect of light condition was found.

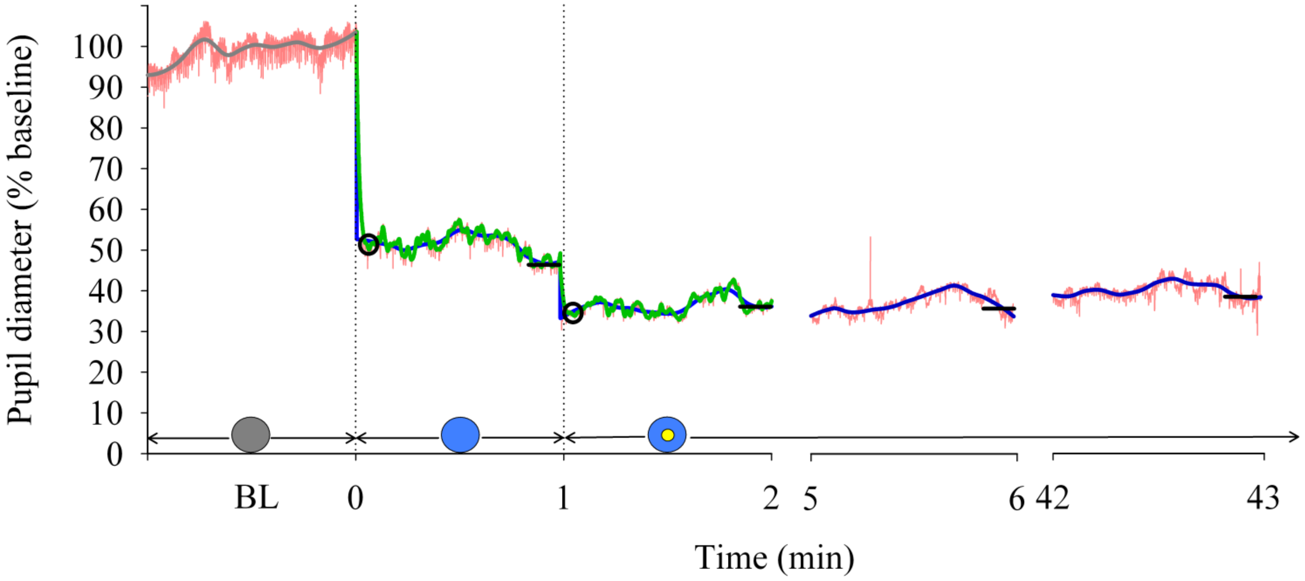

Supplementary Figure 5. Example of individual pupillary constriction, in response to blue-enriched light exposure. The de-artifacted pupil diameter is shown in pink. A smoothing loess function (span 600, grey line) was applied to determine the diameter in dim light baseline condition (BL, grey circle), one minute prior to lights-on. At min 0, the full visual field is exposed to blue-enriched (or red-enriched) light (blue circle). At min 1, a central spot (C1 in this example) is added and maintained until the end of the 50-min light pulse (yellow circle inside blue circle). A smoothing loess function (span 50, green line) was applied to determine the phasic (black circles) response within the first 5 s of each light phase. Another loess function (span 600, blue line) was used to determine the tonic response (black dashes) during the last 10 s of minute 1, 2, 5 and 42. The 2 vertical dotted lines indicate the light transitions, from dim light to blue-enriched (or red-enriched), and to the addition of C1 (or C2).

2 Supplementary Tables

	Minute 1		Minutes 2–50					
	BE	RE	BE-C1	RE-C1	BE-C2	RE-C2	C1	C2
**Photopic**	119	144	455	480	650	676	336	532
**Cyanopic (S-cones)**	298	80	494	276	620	402	196	323
**Melanopic (ipRGCs)**	227	90	475	338	616	479	248	389
**Rhodopic (Rods)**	194	95	447	348	594	495	253	401
**Chloropic (M-cones)**	140	113	439	413	615	589	300	476
**Erythropic (L-cones)**	123	151	462	489	657	686	339	536

Supplementary Table 1. Distribution of photopic and alpha-opic lux content of light exposures. Full-field photopic and alpha-opic content are shown. Content for the C1 and C2 added field sizes are shown separately on the right.

	P value				
**Time-point comparison**	Delta	Theta	High Alpha	Beta	Gamma
1 – 0	-	-	0.0011	0.028	-
1 – 2	0.86	-	0.99	0.75	0.97
1 – 5	0.22	-	0.093	0.86	0.16
1 – 42	0.015	-	0.0082	0.0025	0.36
2 – 5	0.67	-	0.20	0.997	0.05
2 – 42	0.12	-	0.024	0.057	0.65
5 – 42	0.69	-	0.82	0.032	0.0013

Supplementary Table 2. *Post hoc* pairwise comparisons (p-values) between the different time-points for the EEG.

Time-point comparison	P value
1 – 0	-
1 – 2	0.97
1 – 5	0.030
1 - 42	0.0009
2 – 5	0.089
2 - 42	0.0044
5 - 42	0.73

Supplementary Table 3. *Post hoc* pairwise comparisons (p-values) between the different time-points for the DPG.

	P value	
**Time-point comparison**	Heart rate	LF/HF ratio
1 – 0	-	-
1 – 2	0.0098	-
1 – 5	0.031	-
1 – 42	<.0001	-
2 – 5	0.98	-
2 – 42	0.16	-
5 – 42	0.069	-

Supplementary Table 4. *Post hoc* pairwise comparisons (p-values) between the different time-points for heart rate and LF/HF ratio.

**Response**	*a*	*b*	*c*	R^2
Delta	6.39	3.91	2.73	0.95
Beta	9.018	7.64	3.05	0.87
Gamma	29.58	11.49	2.01	0.80
DPG	38.60	3.68	3.32	0.99
PLR	57.67	0.80	5.47	1

Supplementary Table 5. Parameter estimates and R^2 values of fitted duration-response curves for EEG (delta, beta and gamma activity), DPG and the pupillary light reflex (tonic pupil response). A 4-parameter logistic fit was applied. Parameter *a* is the maximum response, *b* is the duration at which 50% of the maximal response is achieved (EC50), *c* is a measure of the steepness of the rising portion of the curve, and *d* is the minimum response and is equal to zero (not shown in table). Goodness of fit is given by the R^2 values.
